# Hypofractionated radiation leads to more rapid bleeding cessation in women with vaginal bleeding secondary to gynecologic malignancy

**DOI:** 10.1186/s13014-022-01995-7

**Published:** 2022-02-14

**Authors:** Luke A. Moradi, Craig S. Schneider, Alok S. Deshane, Richard A. Popple, Robert Y. Kim, Samuel R. Marcrom

**Affiliations:** 1 Department of Radiation Oncology, University of Alabama at Birmingham, Birmingham, AL USA; 2 Case Western Reserve University School of Medicine, Cleveland, OH USA

**Keywords:** Gynecologic cancer, Vaginal bleeding, Radiation, Cervical cancer

## Abstract

**Background:**

Vaginal bleeding (VB) is common in women with gynecologic (GYN) malignancies. Radiation therapy (RT) is used for the definitive treatment of GYN cancers and palliation of bleeding. The historical dogma is that high dose-per-fraction radiation leads to more rapid bleeding cessation, yet there is scant data supporting this claim. We sought to examine the effect of RT fraction size on VB via retrospective analysis of patients receiving hypofractionated radiation (HFRT) compared to conventionally fractionated radiation (CFRT) for control of bleeding secondary to GYN malignancies.

**Methods:**

We identified patients receiving external beam RT for continuous VB from GYN malignancy treated in our department from 2012 to 2020. RT was classified as HFRT (> 2.0 Gy/fx) or CFRT (1.8–2.0 Gy/fx). Demographic information, disease characteristics, and treatment details were collected. The primary endpoint was days from RT initiation until bleeding resolution. Characteristics between groups were compared via Fisher’s exact test. Time to bleeding cessation was assessed via Kaplan–Meier and log-rank test. Univariable and multivariable Cox-proportional hazards were used to identify factors associated with bleeding cessation.

**Results:**

We identified 43 patients meeting inclusion criteria with 26 and 17 patients receiving CFRT and HFRT, respectively. Comparison of baseline characteristics revealed patients receiving HFRT were older (*p* = 0.001), more likely to be post-menopausal (*p* = 0.002), and less likely to receive concurrent chemotherapy (*p* = 0.004). Time to bleeding cessation was significantly shorter for patients receiving HFRT (log-rank *p* < 0.001) with median time to bleeding cessation of 5 days (HFRT) versus 16 days (CFRT). Stratification by dose-per-fraction revealed a dose–response effect with more rapid bleeding cessation with increased dose-per-fraction. While HFRT, age, recurrent disease, prior pelvic RT, and prior systemic therapy were associated with time to bleeding cessation on univariable analysis, HFRT was the only factor significantly associated with time to bleeding cessation in the final multivariable model (HR 3.26, *p* = 0.008).

**Conclusions:**

Patients with continuous VB from GYN tumors receiving HFRT experienced more rapid bleeding cessation than those receiving CFRT. For patients with severe VB, initiation of HFRT to control malignancy related bleeding quickly may be warranted.

**Supplementary Information:**

The online version contains supplementary material available at 10.1186/s13014-022-01995-7.

## Background

Bleeding is a common sequela of gynecologic (GYN) malignancies such as cervical cancer and endometrial cancer and in severe cases can lead to life-threatening anemia requiring transfusions. The pathophysiology behind tumor associated bleeding is initially believed to be secondary to tumor friability, which leads to light bleeding. Later, as the tumor erodes into larger blood vessels, heavier bleeding can develop [1].

Vaginal bleeding (VB) is a common presenting symptom for women with newly diagnosed GYN malignancies, especially endometrial and cervical cancer. Tumor-directed radiotherapy has been shown to be effective in achieving hemostasis in these individuals. There have been multiple studies demonstrating the effectiveness of radiation to induce hemostasis with various fractionation and dosing schemes [2–7], however, there remains no consensus on the optimal radiation schedule for rapid and durable cessation of bleeding. A large proportion of these patients present with curable disease and therefore palliative, hypofractionated RT alone is not appropriate and a more protracted, conventionally fractionated RT course with curative intent is warranted. While there is a prevailing opinion in the radiation oncology community that higher dose-per-fraction RT more rapidly resolves tumor-related bleeding, there is minimal objective data to support this. Further, most published literature has focused on patients undergoing palliative, hypofractionated radiation. While data exists on the effectiveness of conventional fractionation for bleeding cessation as a component of definitive treatment of GYN malignancies, there is a paucity of data evaluating the rate of bleeding cessation with conventional fractionation as compared to hypofractionation. In particular, for curative intent patients (e.g., patients with newly diagnosed locally advanced cervical cancer) with severe bleeding (e.g., requiring frequent transfusions), the optimal radiation regimen for both prompt bleeding cessation and long-term tumor control is not well defined. For example, the merits of starting with a hypofractionated RT course to more rapidly halt bleeding followed later by more protracted, conventional fractionation to a curative intent RT dose (e.g., based on total biologically effective dose, BED) compared to delivering the entirety of the RT course with conventional fractionation is not well understood.

The purpose of this study was to retrospectively compare the efficacy of hypofractionated RT (HFRT, > 2.0 Gy/fraction) to conventionally fractionated RT (CFRT, 1.8–2.0 Gy/fx) for bleeding cessation in women with bleeding secondary to GYN malignancies with inclusion of both palliative and curative intent patients. The goal of this study is to provide a better understanding of the relationship between RT dose and time to bleeding cessation, which may help physicians balance the acute need for bleeding cessation with the need to deliver more curative intent cumulative RT doses.

## Methods

A retrospective analysis was performed on women receiving external beam radiation therapy (EBRT) for continuous VB related to an underlying GYN malignancy at the University of Alabama at Birmingham (UAB) from 2012 to 2020. GYN malignancies included in the study were cervical, endometrial, vaginal, or vulvar. All recorded variables were gathered from UAB electronic medical records (EMR) and the study was approved by UAB’s Institutional Review Board (UAB IRB 120703005).

Basic demographic information such as age, race, menopausal status was collected. Cancer information including primary tumor site (vulvar, vaginal, cervical, or uterine), tumor histology, prior systemic therapy, prior in-field radiation, and prior pelvic surgery was also collected. RT dates, total dose (Gy), total number of fractions delivered, dose-per-fraction, biologically effective dose (α/β = 10 and α/β = 2 or BED_10_ and BED_2_, respectively), subsequent pelvic boost, subsequent brachytherapy, and treatment intent (definitive vs. palliative) were also collected. Patient details during treatment such as concurrent systemic therapy, anticoagulation or antiplatelet use, pre-treatment hemoglobin and platelet count (on first day or within 15 days of treatment start), lowest intra-treatment hemoglobin, post-treatment hemoglobin and platelet count (on final day or within 15 days of final treatment), and whether blood transfusion was needed (during or since cancer diagnosis) were also collected. Patients with intermittent bleeding or spotting were excluded, as well as patients with documented bleeding cessation prior to RT initiation, leaving only patients with continuous bleeding from GYN malignancy. Patients with inadequate documentation of bleeding were excluded, as were patients who received interventional radiology (IR) or surgical intervention to correct VB.

The primary endpoint was days from RT initiation until complete bleeding resolution. Complete bleeding resolution was defined by the first documentation of “no bleeding/bleeding resolved” in the EMR. Documentation of rebleeding, date of rebleeding, and acute toxicities (grade II+) were collected. Acute toxicities (during or within one month following treatment) were graded using Common Terminology Criteria for Adverse Events (CTCAE) v5.0.

Radiation was classified as HFRT (> 2.0 Gy/fx) or CFRT (1.8–2.0 Gy/fx) based on the overall dose-per-fraction from their treatment course. The hypofractionation group was subsequently further sub-stratified into 2.0–2.5 Gy/fraction and > 2.5 Gy/fraction for further investigation of the effect of increasing dose-per-fraction on bleeding cessation. Baseline characteristics between groups were compared via Fisher’s exact test and Mann–Whitney test for categorical and continuous variables, respectively. Time to bleeding cessation was assessed via Kaplan–Meier and log-rank test. Univariable Cox-proportional hazard models was used to identify factors associated with bleeding cessation. Factors with *p* < 0.2 on univariable analysis were subsequently included in a final multivariable Cox-proportional hazards model. Linear regression with Pearson’s correlation was performed to assess the relationship between dose-per-fraction and time to bleeding cessation. The mean BED_10_ and BED_2_ dose delivered (in Gy) at the time of bleeding cessation for HFRT and CFRT was compared with the Mann–Whitney test.

## Results

**Fig. 1:**
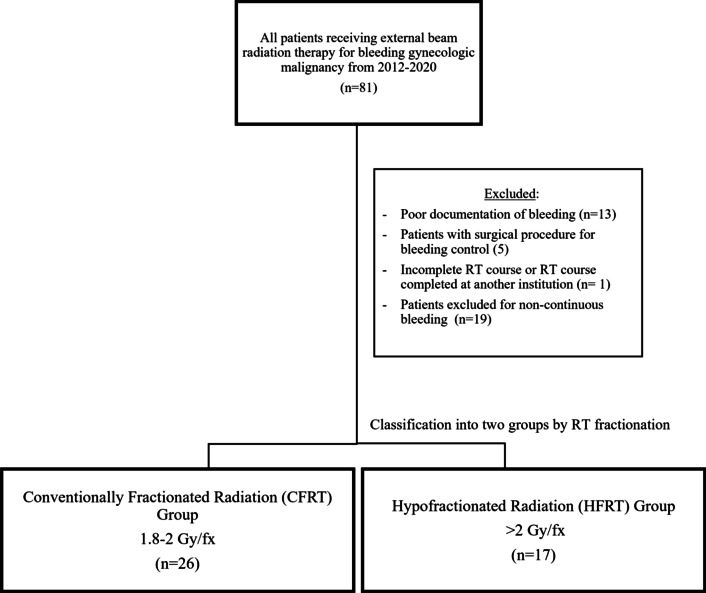
Flow diagram detailing patient inclusion and exclusion criteria for study cohort

We identified 43 patients meeting the pre-determined inclusion/exclusion criteria (Fig. 1). Of those patients, 26 patients received CFRT (1.8–2 Gy/fx) and 17 patients received HFRT (> 2.0 Gy/fx). Comparison of patient characteristics between the groups are shown in Table 1. Patients in the HFRT group were more like to be older, post-menopausal, and have metastatic disease, while patients in the CFRT group were more likely to receive concurrent chemotherapy. Otherwise, there were no statistically significant differences in patient characteristics between the groups.

**Table 1:** Baseline patient characteristics and demographics

Variable	CFRT (N = 26)	HFRT (N = 17)	*P* value
Age (years)			*P* = 0.001
Less than 50	21 (80.7%)	5 (29.4%)	
50 or older	5 (19.3%)	12 (70.6%)	
Race			*P* = 1.000
White	15 (57.7%)	9 (52.9%)	
Non-White	11 (42.3%)	8 (47.1%)	
Primary cancer			*P* = 0.310
Cervical	20 (76.9%)	10 (58.8%)	
Non-Cervical	6 (23.1%)	7 (41.2%)	
Anticoagulation or antiplatelet use			*P* = 1.000
Yes	3 (11.5%)	1 (5.9%)	
No	23 (88.5%)	16 (94.4%)	
Menopausal status			*P* = 0.002
Post-menopausal	7 (26.9%)	13 (76.5%)	
Pre-menopausal	19 (73.1%)	4 (23.5%)	
Transfusion received			*P* = 1.000
Yes	18 (69.2%)	11 (64.7%)	
No	8 (30.8%)	6 (35.3%)	
Concurrent chemotherapy			*P* = 0.004
Yes	21 (80.7%)	6 (35.3%)	
No	5 (19.3%)	11 (64.7%)	
Metastatic disease			
Yes	4 (15.4%)	7 (41.2%)	*P* = 0.007
No	22 (84.6%)	10 (58.8%)	
Recurrent disease			
Yes	2 (7.7%)	4 (23.5%)	*P* = 0.193
No	24 (92.3%)	13 (76.5%)	
Prior pelvic RT			
Yes	0 (0%)	1 (5.9%)	*P* = 0.395
No	26 (100%)	16 (94.1%)	
Prior pelvic surgery			
Yes	5 (19.2%)	6 (35.3%)	*P* = 0.295
No	21 (80.8%)	11 (64.7%)	
Prior systemic therapy			
Yes	0 (0%)	6 (35.3%)	*P* = 0.001
No	26 (100%)	11 (54.7%)	
Pre-treatment hemoglobin	10.00	8.57	*P* = 0.084

The numbers in the parentheses represent the percent of patients within each group. Values reported for continuous variables are the arithmetic mean. *P* values listed were calculated via Fisher’s exact test for categorical variables and Mann–Whitney test for continuous variables

For the CFRT group, all 26 patients received a dosing scheme of 45 Gy in 25 fractions. In the HFRT group, the dosing schemes were varied with regards to total dose received and dose-per-fraction (Table 2). For the CFRT group, the median number of fractions received was 25, while the median number of fractions received in the HFRT group was 15. Biologically effective doses (BED with α/β = 10 or BED_10_) were calculated based on the different dosing schemes within the cohort with median BED_10_ of 53.1 Gy versus 46.9 Gy for CFRT versus HFRT, respectively (*p* = 0.002). A total of 25 patients went on to receive brachytherapy (24 patients in the CFRT and 1 patient in the HFRT group). However, bleeding cessation was achieved in all patients prior to first fraction of brachytherapy.

**Table 2:** External beam radiation regimens utilized in patient cohort. BED10 represents the overall BED calculated with α/β = 10

RT regimen	BED_10_ (Gy)	# (%)	MTTBC (days)
CFRT (N = 26)			
1.8 Gy × 25 fx = 45 Gy	53.1	26 (100%)	16 [6–41]
HFRT (N = 17)			
2.5 Gy × 16 fx = 40 Gy	50.0	4 (24%)	15.5 [5–28]
2.5 Gy × 15 fx = 37.5 Gy	46.9	4 (24%)	11 [5–17]
2.5 Gy × 10 fx = 25 Gy	31.3	1 (6%)	13
1.8 Gy × 1 fx − > 3 Gy × 3 fx − > 1.8 Gy × 20 fx = 46.8 Gy total^a^	56.3	1 (6%)	1
2.2 Gy × 20 = 44 Gy	53.7	1 (6%)	6
4 Gy × 2 − > 3 Gy × 10 fx = 38 Gy total	50.2	1 (6%)	4
4 Gy × 3 fx − > 3 Gy × 4 fx = 24 Gy total	32.4	1 (6%)	1
4Gy × 5 = 20 Gy	28.0	1 (6%)	1
3.7Gy × 4 = 14.8 Gy delivered BID	20.3	1 (6%)	1
3.7 Gy × 4 = 14.8 Gy	20.3	1 (6%)	1
2.5 Gy × 3 = 7.5 Gy^b^	9.4	1 (6%)	2

^a^Initially planned for 1.8 Gy × 25 = 45 Gy but had worsening bleeding so switched to 3 Gy × 3 fx = 9 Gy, later followed by completion of definitive RT 36 Gy in 20 fx

^b^Patient planned for 2.5 Gy × 16 fx = 40 Gy but decided to go on hospice and stopped RT

Patients with multiple fractionation schemes delivered sequentially are denoted with forward arrow sign (− >). Median time to bleeding cessation (MTTBC) is reported in days with number in brackets representing the range for fractionation schemes with multiple patients

**Fig. 2:**
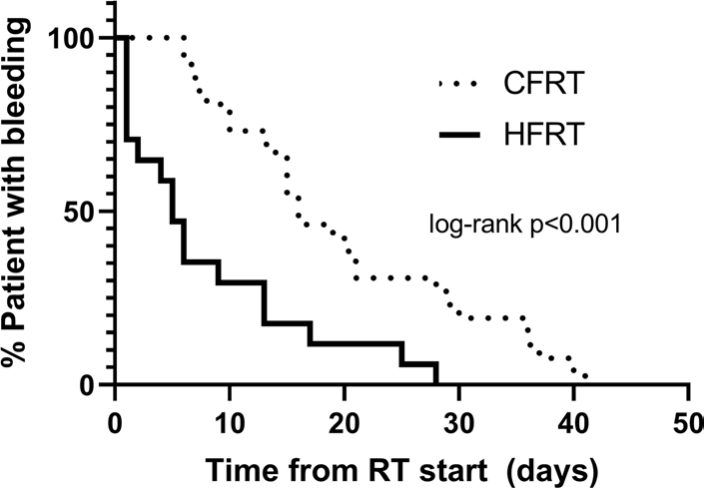
The effect of radiation type on time to bleeding cessation. Time to bleeding cessation from initiation of first fraction of radiation is presented via Kaplan–Meier analysis for patients receiving conventionally fractionated radiation therapy (CFRT) and hypofractionated radiation therapy (HFRT). Log-rank *p* value was used to compare time-to-bleeding cessation between the groups (*p* < 0.001). Median time to bleeding cessation was 16 days and 5 days for CFRT and HFRT, respectively

To examine differences in time to bleeding cessation between the groups, Kaplan–Meier analysis and log-rank testing were performed (Fig. 2). Median time to bleeding cessation was 5 days for those receiving HFRT compared to 16 days for those receiving CFRT. Log-rank testing confirmed more rapid time to bleeding cessation for the patients receiving HFRT compared to those receiving CFRT (log rank *p* < 0.001).

To identify other factors that might contribute to differences in time to bleeding cessation, cox univariable regression was performed (Table 3) with a pre-determined p-value cut point of *p* < 0.2 set for inclusion in a final multivariable model. On univariable analysis, RT type (HFRT vs. CFRT, HR = 3.23, *p* < 0.001) age (< 50 years old vs. 50+ years old, HR = 2.17, *p* = 0.022), recurrent disease (recurrent disease vs. newly diagnosed, HR 3.53, *p* = 0.007), prior pelvic RT (yes vs. no, HR = 10.50, *p* = 0.035), and prior systemic therapy (yes vs. no, HR 6.12, *p* < 0.001) were significantly associated with time to bleeding cessation. However, in the final multivariable cox proportional hazards model, only RT type (HFRT vs. CFRT) was significantly associated with time to bleeding cessation (HR 3.26, *p* = 0.008).

**Table 3:** Univariable and multivariable cox proportional hazards analysis of the association of listed dichotomized variables with time to bleeding cessation

Variable	Univariable analysis			Multivariable analysis		
	HR	95% CI	*P* value	HR	95% CI	*P* value
CFRT	3.23	1.65–6.32	0.001	3.26	1.37–7.80	0.008
Age 50+	2.17	1.11–4.22	0.022	0.74	0.28–1.96	0.543
Concurrent chemo	0.54	0.29–1.02	0.057	0.76	0.31–1.88	0.550
Non-White	0.97	0.53–1.80	0.941			
AC or AP use	0.62	0.22–1.77	0.373			
Non-cervical primary	1.20	0.62–2.33	0.581			
Pre-menopausal	0.70	0.38–1.29	0.259			
Transfusion required	1.14	0.60–2.21	0.680			
Metastatic	1.15	0.60–2.20	0.669			
Recurrence	3.53	1.42–8.82	0.007	1.96	0.51–7.54	0.326
Prior pelvic RT	10.50	1.17–93.9	0.035	2.06	0.16–27.00	0.582
Prior pelvic surgery	1.87	0.93–3.77	0.080	1.76	0.68–4.57	0.243
Prior systemic therapy	6.12	2.30–16.33	< 0.001	2.17	0.64–7.28	0.211
Pretreatment Hgb (continuous)	0.96	0.82–1.12	0.597			

Pre-specified criteria for inclusion in a final multivariable model was *p* < 0.2 on univariable analysis

**Fig. 3:**
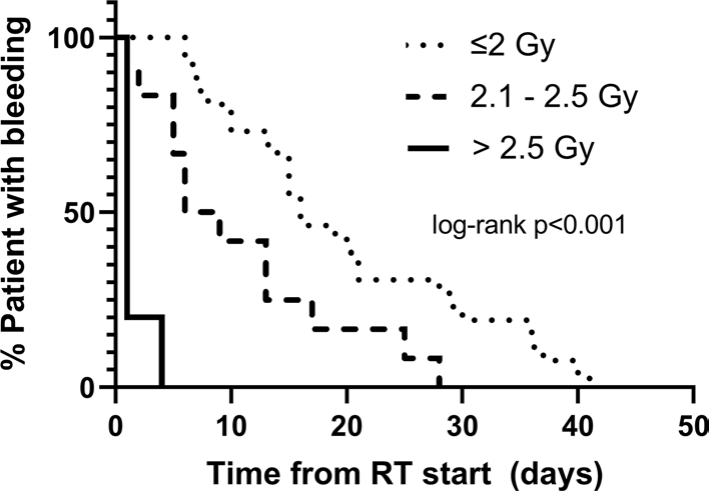
The effect of radiation dose per fraction on time to bleeding cessation. Time to bleeding cessation is presented for patients receiving less than 2.0 Gy per fraction (≤ 2.0 Gy/fx), 2.1–2.5 Gy per fraction (2.1–2.5 Gy/fx) and more than 2.5 Gy per fraction (> 2.5 Gy/fx). Log-rank *p* value was calculated at *p* < 0.001. Log-rank *p* testing between each individual group pairing was tested and yielded *p* = 0.2457 for the low dose compared to intermediate dose per fraction (≤ 2.0 Gy/fx vs. 2.1–2.5 Gy/fx) but comparison of high dose per fraction (> 2.5 Gy/fx) with the other two groups yielded *p* < 0.001 on log-rank p-testing

To further investigate the effect of more hypofractionated RT courses (with increasing dose-per-fraction) on time to bleeding cessation, we performed a subset analysis of time to bleeding cessation where the HFRT group was further divided based on dose-per-fraction (2.0–2.5 Gy/fraction vs. > 2.5 Gy/fraction). The Kaplan–Meier results of this analysis are shown in Fig. 3 and shows an apparent dose response effect with rapid bleeding cessation for the highest dose cohort (> 2.5 Gy/fraction). In fact, all patients receiving more than 2.5 Gy/fraction had bleeding cessation within 4 days of initiation of RT and median time to bleeding cessation of 1 day. Overall, median time to bleeding cessation decreased with increasing dose-per-fraction with median values of 16 days, 6 days, and 1 day for dose-per-fraction values of ≤ 2.0 Gy, 2.1–2.5 Gy, and > 2.5 Gy, respectively. Linear regression analysis of time to bleeding cessation versus dose-per-fraction demonstrated a significant inverse correlation (Additional file 1: Figure S1, r = − 0.4684, *p* = 0.0015).

**Fig. 4:**
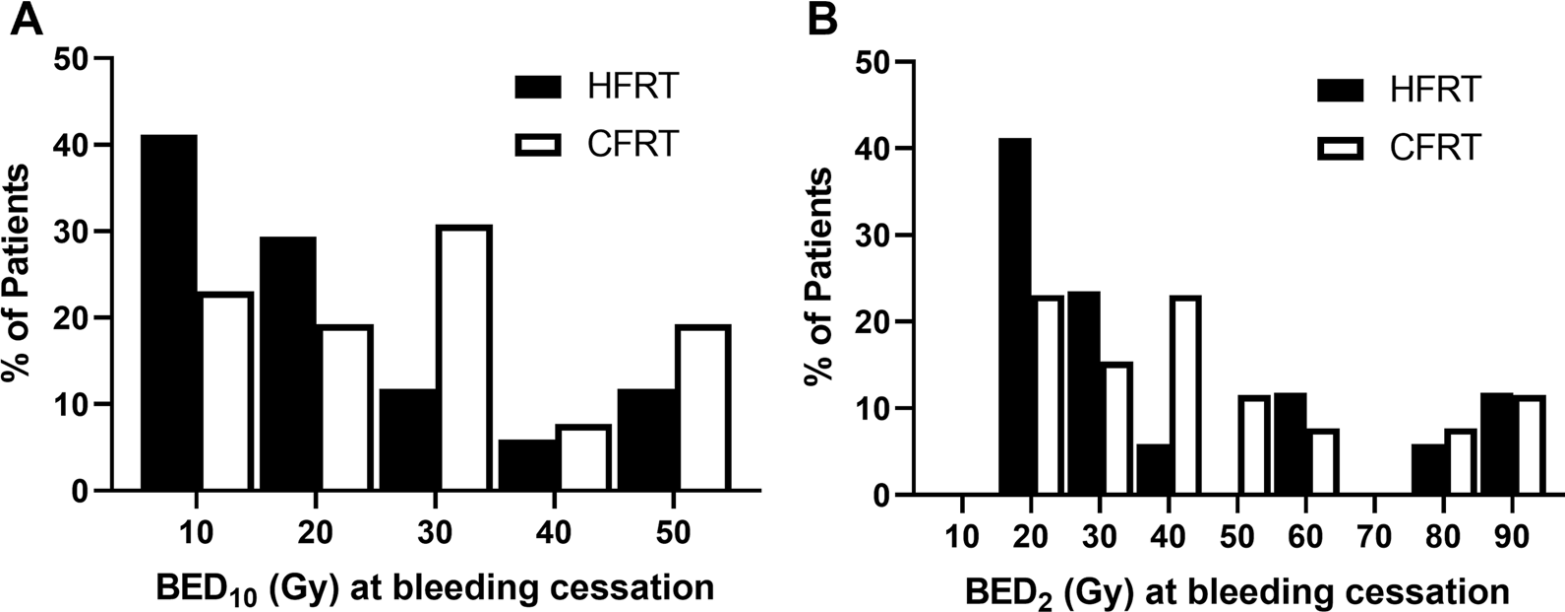
Histogram showing the cumulative dose in **A** BED10 or **B** BED2 delivered at the time bleeding cessation was documented for patients receiving CFRT (white bars) and HFRT (black bars). Median BED10 at time of bleeding cessation was 25.5 Gy and 15.2 Gy for CFRT and HFRT, respectively (*p* = 0.041 by Mann–Whitney test). Median BED2 at time of bleeding cessation was 41.0 Gy and 28.1 Gy for CFRT and HFRT, respectively (*p* = 0.269 by Mann–Whitney test)

To investigate the relationship between BED_10_ dose delivered to the tumor and bleeding cessation, we plotted histograms of the cumulative BED_10_ dose delivered at the time of bleeding cessation for individual patients in each group (Fig. 4). For the whole cohort, the median BED_10_ delivered at the time of bleeding cessation was 21.9 Gy. Patients receiving HFRT had bleeding cessation at lower total delivered BED_10_ doses compared to those in the CFRT group with median values of 15.2 Gy for HFRT compared to 25.5 Gy for CFRT (*p* = 0.041). We also calculated BED_2_ (biologically effective dose at α/β = 2) dose delivered at the time of bleeding cessation to estimate the biologically effective dose to tumor-associated endothelial cells (assuming normal endothelial tissue α/β = 2). For the entire cohort, the median BED_2_ dose at time of bleeding cessation was 37.6 Gy. While the median BED_2_ dose at time of bleeding cessation was lower for HFRT compared to CFRT (28.1 Gy vs. 41.0 Gy), this difference was not statistically significant (*p* = 0.269).

We additionally looked at overall bleeding control and rates of rebleeding in our patient population. All patients in the study had cessation of bleeding with bleeding control rates of 100%, irrespective of RT type (CFRT vs. HFRT). In all cases, bleeding cessation was achieved with external beam RT and prior to initiation of any brachytherapy. A total of 4 patients (9.3%) within the cohort experienced rebleeding after initial bleeding resolution with a median time to rebleeding of 128 days (range 48–216 days). Of those four patients, two received HFRT and 2 received CFRT, resulting in rebleeding rates of 7.7% and 11.4%, respectively (χ^2^ *p* = 0.653). The rate of acute grade 2+ toxicity was higher in patients receiving CFRT compared to HFRT (61.5% vs. 23.5%, *p* = 0.027) with upper GI (nausea), lower GI (diarrhea), and GU (dysuria) the most common toxicities observed (Additional file 2: Table S2). Grade 3+ toxicity was rare with two patients experiencing Grade 3 upper GI toxicity in the CFRT group and one patient experiencing Grade 3 bladder outlet obstruction (secondary to blood clot from tumor invasion of the bladder) in the HFRT group. Pre- and post-RT hemoglobin and platelet counts for patients with available data, as well as the lowest hemoglobin level recorded during RT treatment are shown in Additional file 1: Figure S1. There was a trend towards a lower pre-RT hemoglobin for patients receiving HFRT compared to CFRT (10.00 vs. 8.57, *p* = 0.084), but, otherwise, no difference in any of the other parameters.

## Discussion

Despite being a common issue encountered in radiation oncology, the optimal RT regimen for palliation of VB and implications of RT fractionation scheme on time to bleeding cessation are not well defined. We found that women receiving HFRT had significantly decreased time to bleeding cessation compared to CFRT with median time to bleeding cessation of 5 days versus 16 days, respectively. When HFRT was further subdivided by the amount of dose delivered per fraction, the data continued to demonstrate that increasing dose-per-fraction decreased time to bleeding cessation with an apparent dose–response effect. On multivariable analysis, receipt of HFRT (as opposed to CFRT) was the only variable with a statistically significant association with time to bleeding cessation. Altogether, these data suggest that in our cohort of patients, more hypofractionated radiation regimens leads to more rapid bleeding cessation in patients with tumor-associated VB.

While the main result of this study, that higher dose-per-fraction RT leads to more rapid tumor-related bleeding cessation, seems somewhat intuitive and is a commonly held belief by radiation oncologists, there is a paucity of modern data supporting this point. The limited studies that have investigated the relationship between radiation dose and GYN bleeding cessation have focused on incurable, palliative intent patients and in many cases, very hypofractionated RT regimens (e.g., 10 Gy in a single fraction) [2, 3, 5, 6]. Further, most of these studies reported only overall response rates but no information regarding how quickly bleeding cessation was achieved. Other studies lumped patients with bleeding GYN malignancies together with other bleeding tumor sites (e.g., bladder/hematuria, lung/hematemesis, etc.), making interpretation of the combined results challenging [4]. One of the few contemporary studies that focused specifically on patients with bleeding GYN malignancies following palliative RT reported similar overall findings to those reported here [8]. While they did not find a statistically significant difference in the time to bleeding cessation in their small cohort of incurable patients receiving palliative RT, time to bleeding cessation was numerically less with more hypofractionated RT and with similar times to bleeding cessation compared to those reported here.

Our study is somewhat unique in that we have included both patients treated with curative and palliative intent radiation. The data presented here provides information on bleeding cessation with more protracted RT regimens and gives useful insight into the relative effectiveness and kinetics of bleeding cessation for more curative-intent RT compared to shorter-course hypofractionated RT. For example, this data may be helpful in the management of patients with newly diagnosed, curable cervical cancer with severe bleeding at presentation. If a patient presents with severe bleeding at presentation (e.g., requiring frequent transfusions), initiation of RT with a few fractions of HFRT may be warranted before switching to a more conventionally fractionated definitive RT course, as our data suggests a significant reduction in median time to bleeding cessation (5 days vs. 16 days) with HFRT. Alternatively, patients with less severe bleeding may be managed appropriately with CFRT with expected bleeding cessation most likely within approximately 2 weeks of RT initiation. Several studies have found an association between anemia and worse oncologic outcomes in cervical cancer patients receiving radiation, possibly related to tumor hypoxia leading to increased radioresistance [9–12]. While the exact mechanism for this association is not well understood, these data suggest that more rapid cessation of bleeding may limit or prevent anemia and in turn improve oncologic outcomes, which may also be a consideration when choosing RT dose and strategy.

As we did include both curative and palliative intent patients with bleeding GYN malignancy, we must acknowledge that patients receiving CFRT and HFRT were inherently different. Patients being treated with curative intent CFRT were more likely to be younger, treatment naïve patients with cervical cancer and were more likely to receive concurrent chemotherapy, whereas patients receiving HFRT were more likely to be older patients with non-cervical malignancy with recurrent disease. Further, physicians likely elected to use HFRT for patients with more severe bleeding. This is consistent with the lower pre-RT hemoglobin levels seen in the HFRT group compared to CFRT (10.00 g/dL vs. 8.57 g/dL, *p* = 0.084). Taking all these facts together, patients in the HFRT group likely had more severe bleeding and more recalcitrant tumors, making the significant reduction in time to bleeding cessation between HFRT and CFRT even more meaningful.

Another unique aspect of our study is that we calculated the individual BED_10_ and BED_2_ doses that were delivered for individual patients at the time of physician-documented bleeding cessation. The goal of this analysis was to provide physicians a tool to estimate time to bleeding cessation for various RT fractionation schemes (via calculation of BED_2_ and BED_10_) when considering various regimens for patients with active bleeding. We found that the BED_10_ dose delivered at time of bleeding cessation was statistically significantly greater for CFRT compared to HFRT. However, the BED_2_ dose delivered at time of bleeding cessation was not statistically different. This suggests that time to initial bleeding cessation may be more driven by the effect of RT on tumor associated endothelium (with lower associated α/β) rather than on tumor cells. Indeed, there is some data to suggest that more hypofractionated RT doses may more greatly impact/damage tumor-associated endothelium, which may have led to earlier bleeding cessation for patients receiving HFRT in this study [13–15].

There are several limitations to the present study. Given the retrospective nature, there are limitations regarding the granularity of bleeding cessation data that was obtained. Despite this, we still saw a statistically significant difference in time to bleeding cessation for CFRT versus HFRT, and our median time to bleeding cessation and bleeding control rates were similar to those reported in contemporary studies as discussed above. Additionally, assessment of bleeding cessation was reliant on the somewhat subjective measure of physician documentation of bleeding cessation. While we did collect more objective data related to bleeding, such as hemoglobin levels and need for blood transfusion, being a retrospective study, not all patients had this data available and it was also confounded by other factors, such as chemotherapy induced anemia. Given these limitations, it was not feasible to use these data as a more objective endpoint for bleeding cessation/control. While our cohort size of 43 patients is quite sizeable compared to similar analyses in the literature, from a statistical standpoint this is a relatively small sample size with limited power and risk of type 2 error. Despite this, we did still see significant differences in time to bleeding cessation with RT regimen choice. However, other variables that one might expect to influence bleeding cessation, such as receipt of anticoagulant or antiplatelet medications, were not significantly associated with time to bleeding cessation, possibly due to the limited sample size and power.

## Conclusion

Women with continuous VB from GYN malignancies receiving HFRT experienced more rapid bleeding cessation than those receiving CFRT with an apparent dose response effect with increasing dose-per-fraction. For patients with severe GYN bleeding, initiation of high dose-per-fraction radiation to rapidly halt malignancy related bleeding may be warranted.

## Supplementary Information

**Additional file 1.** **Supplemental Figures: Supplemental Figure 1.** Relationship between dose per day delivered and time to bleeding cessation. Line shown is simple linear regression demonstrating an inverse correlation (Pearson’s correlation r = -0.4684, p = 0.0015)

**Additional file 2.** **Supplemental Tables: Supplemental Table 1.** Hemoglobin and platelet levels before and after completion of radiation. Values reported are the arithmetic mean. P-values were calculated by Mann-Whitney test. **Supplemental Table 2** Acute toxicities reported by RT type received. Grade 2+ toxicities per CTCAE criteria are reported. P-values were determined via Fisher’s exact test.

## Data Availability

The datasets used and/or analysed during the current study are available from the corresponding author on reasonable request.

## Abbreviations

VB: Vaginal bleeding
GYN: Gynecologic
RT: Radiation therapy
HFRT: Hypofractionated radiation
CFRT: Conventionally fractionated radiation
EBRT: External beam radiation therapy
EMR: Electronic medical record
BED: Biologically effective dose
CTCAE: Common Terminology Criteria for Adverse Events
HR: Hazard ratio
IR: Interventional radiology
