# Supplementary figures and images for: Hypofractionated radiation leads to more rapid bleeding cessation in women with vaginal bleeding secondary to gynecologic malignancy
Source: Radiat Oncol. 2022 Feb 14;17:34. doi: 10.1186/s13014-022-01995-7 (PMC8842901; doi:10.1186/s13014-022-01995-7)

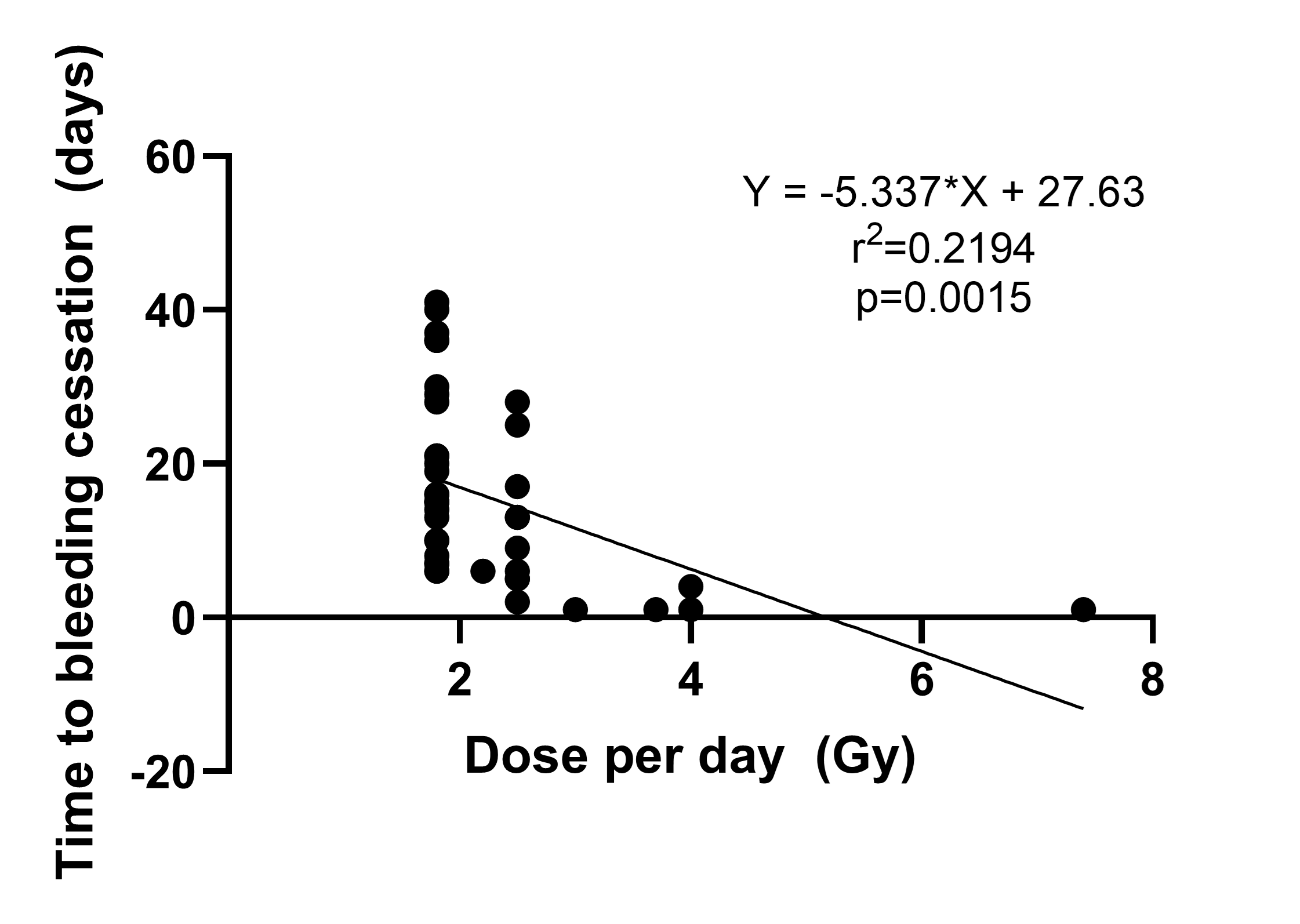

Supplement: Supplementary file 1 — Additional file 1. Supplemental Figures: Supplemental Figure 1. Relationship between dose per day delivered and time to bleeding cessation. Line shown is simple linear regression demonstrating an inverse correlation (Pearson’s correlation r = -0.4684, p = 0.0015) [file 13014_2022_1995_MOESM1_ESM.tif]
