# Supplementary material for: Hypofractionated radiation leads to more rapid bleeding cessation in women with vaginal bleeding secondary to gynecologic malignancy
Source: Radiat Oncol. 2022 Feb 14;17:34. doi: 10.1186/s13014-022-01995-7 (PMC8842901; doi:10.1186/s13014-022-01995-7)
Supplement: Supplementary file 2 — Additional file 2. Supplemental Tables: Supplemental Table 1. Hemoglobin and platelet levels before and after completion of radiation. Values reported are the arithmetic mean. P-values were calculated by Mann-Whitney test. Supplemental Table 2 Acute toxicities reported by RT type received. Grade 2+ toxicities per CTCAE criteria are reported. P-values were determined via Fisher’s exact test. [file 13014_2022_1995_MOESM2_ESM.docx]

**Supplemental Tables and Figures**

Tables

**Supplemental Table 1**: Hemoglobin and platelet levels before and after completion of radiation. Values reported are the arithmetic mean. P-values were calculated by Mann-Whitney test.

| **Variable** | **CFRT** | **HFRT** | **P-value** |
| --- | --- | --- | --- |
| Pre-treatment hemoglobin (g/dl) | 10.0 | 8.57 | 0.084 |
| Pre-treatment platelets (g/dl) | 316.8 | 290.1 | 0.077 |
| Post-treatment hemoglobin (g/dl) | 9.18 | 9.42 | 0.588 |
| Post-treatment platelets (g/dl) | 236.7 | 307.3 | 0.414 |
| Lowest Hemoglobin (g/dl) | 7.98 | 8.26 | 0.667 |

**Supplemental Table 2** – Acute toxicities reported by RT type received. Grade 2+ toxicities per CTCAE criteria are reported. P-values were determined via Fisher’s exact test.

| **Toxicity** | **CFRT** | **HFRT** | **P-value** |
| --- | --- | --- | --- |
| Any Grade 2+ toxicity | 61.5% | 23.5% | 0.027 |
| Grade 2+ Upper GI toxicity | 26.9% | 17.7% | 0.714 |
| Grade 2+ Lower GI toxicity | 34.6% | 5.9% | 0.061 |
| Grade 2+ GU toxicity | 19.2% | 5.9% | 0.376 |
| Grade 2+ skin toxicity | 3.9% | 0.0% | 1.000 |

Figures

**Supplemental Figure 1**: Relationship between dose per day delivered and time to bleeding cessation. Line shown is simple linear regression demonstrating an inverse correlation (Pearson’s correlation r=-0.4684, p=0.0015)
